# Hydrogen Cyanide Generation by *Pseudomonas aeruginosa* Blunts the Host Innate Immune Response

**DOI:** 10.1093/infdis/jiag244

**Published:** 2026-05-06

**Authors:** Csaba Szabo, Sidnéia Sousa Santos, Jacqueline Findlay, Olivier Bremer, Maria Petrosino, Thilo Magnus Philipp, Anna Kierońska-Rudek, Karim Zuhra, Gerry R Boss, Patrice Nordmann

**Affiliations:** Section of Pharmacology, Department of Oncology, Microbiology and Immunology, University of Fribourg, Fribourg, Switzerland; Section of Pharmacology, Department of Oncology, Microbiology and Immunology, University of Fribourg, Fribourg, Switzerland; Section of Microbiology, Department of Oncology, Microbiology and Immunology, University of Fribourg, Fribourg, Switzerland; Section of Pharmacology, Department of Oncology, Microbiology and Immunology, University of Fribourg, Fribourg, Switzerland; Section of Pharmacology, Department of Oncology, Microbiology and Immunology, University of Fribourg, Fribourg, Switzerland; Section of Pharmacology, Department of Oncology, Microbiology and Immunology, University of Fribourg, Fribourg, Switzerland; Section of Pharmacology, Department of Oncology, Microbiology and Immunology, University of Fribourg, Fribourg, Switzerland; Section of Pharmacology, Department of Oncology, Microbiology and Immunology, University of Fribourg, Fribourg, Switzerland; Department of Medicine, University of California, San Diego, La Jolla, California, USA; Section of Microbiology, Department of Oncology, Microbiology and Immunology, University of Fribourg, Fribourg, Switzerland

**Keywords:** cyanide, macrophage, sepsis, infection, bioenergetics

## Abstract

**Background:**

The biological mediator hydrogen cyanide (HCN) is produced by certain pathogenic bacteria. Hydrogen cyanide exerts multiple effects in mammalian cells: at low concentrations, it has cytoprotective regulatory effects, whereas at high concentrations, it blocks mitochondrial electron transport by inhibiting cytochrome c oxidase, thereby halting cellular metabolism. Here, we evaluated whether bacterial HCN confers resistance to the host immune response. We used *Pseudomonas aeruginosa* as a model organism, since it is a clinically significant HCN-generating bacterial species and a common cause of nosocomial infections.

**Methods:**

The study used bacterial mutants, macrophage co-cultures, cyanide quantification, phagocytosis and bioenergetics assays, and mouse infection models to assess HCN's role in immune evasion.

**Results:**

Compared with wild-type *P. aeruginosa*, genetically HCN-deficient bacteria were more susceptible to killing by immune cells in vitro and were cleared more rapidly in mouse models of systemic infection. Increased leukocyte killing was not due to increased phagocytosis. Pharmacological scavenging of HCN also enhanced leukocyte bacterial killing.

**Conclusions:**

These findings support the concept that HCN acts as a bacterial defense mechanism against the host's immune response. Targeting bacterial HCN could be a potential therapeutic strategy to improve the immune clearance of *P. aeruginosa* infections.

## Teaser

Hydrogen cyanide produced by *Pseudomonas aeruginosa* protects bacteria by counteracting host-mediated killing. Cyanide-deficient mutants or cyanide scavenging increased leukocyte-mediated clearance in vitro and in vivo, identifying bacterial cyanide generation as an immune evasion mechanism and potential therapeutic target.

Hydrogen cyanide (HCN) has recently emerged as an endogenous gaseous biological mediator that regulates metabolism and cell viability in mammalian cells and tissues. Hydrogen cyanide is generated at low levels from glycine via a lysosomal pathway [1, 2]. Low concentrations of cyanide can stimulate mitochondrial respiration and confer cytoprotective effects [1, 3, 4] while disruptions in HCN homeostasis are associated with suppressed mitochondrial function and impaired cellular viability [1, 2, 5].

Bacteria also produce HCN from glycine, via the bacterial enzyme HCN synthase [6–8]. Hydrogen cyanide released by bacteria can diffuse into the environment and adversely affect other organisms. For example, *Pseudomonas*-derived cyanide serves as a bacterial “weapon” to suppress the growth of competing, co-colonizing bacteria [9, 10]. Moreover, in *Pseudomonas aeruginosa—C*aenorhabditis *elegans* co-cultures, bacterially generated HCN impaired the motility and viability of the nematodes [11, 12]. Similarly, human *P. aeruginosa* isolates that produce cyanide were toxic to *Drosophila melanogaster* in co-culture, largely due to their HCN production [13]. Finally, recent studies demonstrate that supernatants from *P. aeruginosa* cultures can suppress mitochondrial respiration in mammalian cells by inhibiting complex IV [4].

We hypothesized that HCN production might protect bacteria from the host immune response and addressed this question by manipulating bacterial HCN levels without altering host HCN production or elimination. The selected strain was *P. aeruginosa* strain PAO1, a widely used, moderately virulent laboratory reference strain for studying the biology, genetics, and pathogenesis of this bacterium.

## METHODS

### Bacterial Characterization and Culture

Isogenic strains, *P. aeruginosa* PAO1 (ATCC 15692; wild-type or WT) and PAO1 TnhcnB (Δ*hcn*) were obtained from the “PAO1 transposon mutant library” (https://www.gs.washington.edu/labs/manoil/libraryindex.htm), with gene hcnB being disrupted by a transposon insertion. The hcnB transposon disruption was confirmed by whole-genome sequencing, performed on an Illumina NextSeq 1000 instrument. In addition, quality control polymerase chain reactions (PCRs) were performed throughout the study using primers Check_F (5′-GGTGGAAACCGAAAGCAACC-3′) and Check_R (5′-CCTGGACAGTTGGTAGGCG-3′), to ensure the maintenance of the transposon insertion. Wild-type bacteria were cultured on LB agar and Δ*hcn* bacteria were cultured on LB agar with 60 mg/L tetracycline for 18–24 hours at 37°C. One or more colonies were collected, diluted in saline to make a 0.5 McFarland suspension, before dilution to the desired cell concentration. The colony-forming unit (CFU)/mL cell counts were additionally controlled by the plating of serial dilutions of the 0.5 McFarland suspension. Approximately 1 × 10^7^ bacteria were incubated with 10% fetal bovine serum (FBS) for 30 minutes at 37°C for opsonization.

### Cell Culture and Differentiation Protocol

The human pro-monocytic leukemia cell line U937 (ATCC # CRL-1593.2) was grown in RPMI 1640 medium supplemented with 10% FBS, 100 units/mL penicillin G, 100 μg/mL of streptomycin, and 2 mM L-glutamine. Cells were differentiated with phorbol myristate acetate (PMA) 40 ng/mL for 48 hours in a 5% CO_2_ incubator at 37°C. Cells at passages 4–10 were used. The J774A.1 murine macrophage cell line (ATCC® TIB-67™) was cultured in Dulbecco‘s Modified Eagle Medium (DMEM) supplemented with 10% FBS and 1% antibiotic-antimycotic.

### Immunophenotyping and Cell Viability Assessment

The expression of cell surface receptors was investigated to determine the cell profile after exposure to PMA. U937 cells (0.3 × 10^6^) were transferred to polystyrene tubes and stained with the following monoclonal antibodies (activation and viability markers): anti-CD11b-BV421, anti-CD86-BV605, anti-CD80-PE (Biolegend, San Diego, CA, United States), anti-CD14-BV711 and 7-AAD (BD Biosciences) and analyzed by flow cytometry.

### Determination of Intracellular Cytokine Levels

U937 cells were incubated with LPS (100 ng/mL) in nonadherent tubes for 30 minutes. Brefeldin was added (10 µg/mL) and cells were incubated in a 5% CO_2_ incubator at 37°C for 5 hours. Surface and intracellular cytokine labeling was performed by flow cytometry via a BD kit for fixation and permeabilization using anti-human-IL-6-APC (558276) and anti-human-TNF-α (558273) antibodies.

### Detection of Cyanide

Cyanide concentration in the supernatants was determined using a cyanide-selective electrode (Lazar Research Labs) as described [1] or via the Feigl–Anger paper method as described [14].

### Bacterial Killing Assay

To determine the influence of HCN on the microbicidal ability of macrophages, opsonized WT or Δ*hcn* bacteria were pre-incubated in the absence or presence of cells (MOI 1) in a 5% CO_2_ atmosphere at 37°C for 3 hours. Cells were lysed with 500 μL of 0.2% Triton X-100, sonicated for 2 minutes, and kept at room temperature for 15 minutes. The samples were diluted, seeded in Petri dishes containing LB agar, and incubated at 37°C. Colony number was enumerated as CFU/mL after 24 hours. To evaluate the influence of cyanide on the effectiveness of bacteria elimination by U937-derived macrophages, experiments were performed under the same conditions in the presence of vitamin B_12_ (hydroxocobalamin) (Sigma) at 30 or 100 µM or trihistidyl cobinamide (THC) at 30 µM.

### Phagocytosis Assay

Influence of HCN-producing bacteria on phagocytosis capacity was evaluated in U937-derived macrophages co-cultured with WT or Δ*hcn* bacteria labeled with green fluorescent protein (GFP), delivered via a plasmid. Cells (1 × 10^6^) were distributed in nonadherent tubes and incubated in the absence or presence of WT or Δ*hcn Pseudomonas* (MOI 1) in a 5% CO_2_ incubator at 37°C for 3 hours. After incubation, cells were suspended in fluorescence-activated cell sorting (FACS) buffer and centrifuged at 800*g* for 5 minutes at 4°C. The cells were suspended in FACS buffer for further analysis by flow cytometry. The data were plotted as a percentage of cells positive for GFP. We also confirmed the phagocytosis capacity of U937-derived macrophages using pHrodo™ Green *Escherichia coli* BioParticles™ conjugated to fluorescein isothiocyanate.

### Confocal Microscopy

U937-derived macrophages, prepared in parallel with the conditions used for the flow cytometry–based phagocytosis assay, were plated in 4-chamber glass-bottom dishes and incubated for 3 hours at 37°C. Next, cells were gently washed 3× using Hank′s Balanced Salt Solution (HBSS). Representative pictures were collected using a Leica SP5 microscope equipped with a 100× oil-immersion objective.

### MTT Assay

The 3-[4,5-dimethylthiazol-2-yl]-2,5 diphenyl tetrazolium bromide (MTT) assay was performed, as described [15].

### Measurement of Cellular ATP Content

The measurement of cellular ATP content was conducted via the CellTiter-Glo® Luminescent Cell Viability Assay (Promega), according to the manufacturer's instructions.

### In Vivo Experiments

Adult female C57BL/6J mice (10 weeks old; Janvier Labs) were used. Procedures were carried out in accordance with the Swiss Federal Animal Welfare Act of 16 December 2005. One day before infection, WT and Δ*hcn* bacteria were plated on LB agar and incubated at 37°C overnight to obtain fresh cultures. Bacteria were pelleted by centrifugation at 8000 rpm (7.1 *g*) for 10 minutes, washed and resuspended in fresh sterile 0.85% NaCl, and kept on ice until use. Each mouse was inoculated intraperitoneally with 400 μL of the bacterial suspension. In the first series of experiments, 1 × 10^8^ CFU was delivered per animal and blood and spleen were collected at 8 hours postinfection. This relatively early time point was selected, as it is commonly used in acute infection and early host–pathogen interaction studies, particularly in systemic infection models, to capture early bacterial clearance and host response dynamics before secondary complications arise. In a set of follow-up experiments, an identical protocol was performed, with reduced bacterial load (1 × 10^6^ CFU). Since preliminary experiments indicated that at this lower bacterial load, blood CFUs will no longer be detectable; in these experiments, spleen and liver were collected. Tissues were homogenized and serially diluted and plated on LB agar. Plates were then incubated at 37°C for 2 days, and CFUs were counted as described above.

### Statistical Analysis

Data are presented as mean ± standard error of the mean (SEM). Comparisons among multiple groups were performed using analysis of variance, while comparisons between 2 groups were evaluated with Student's *t*-tests (paired or unpaired, as appropriate). A *P*-value of <.05 was considered statistically significant.

## RESULTS

**Figure 1.**
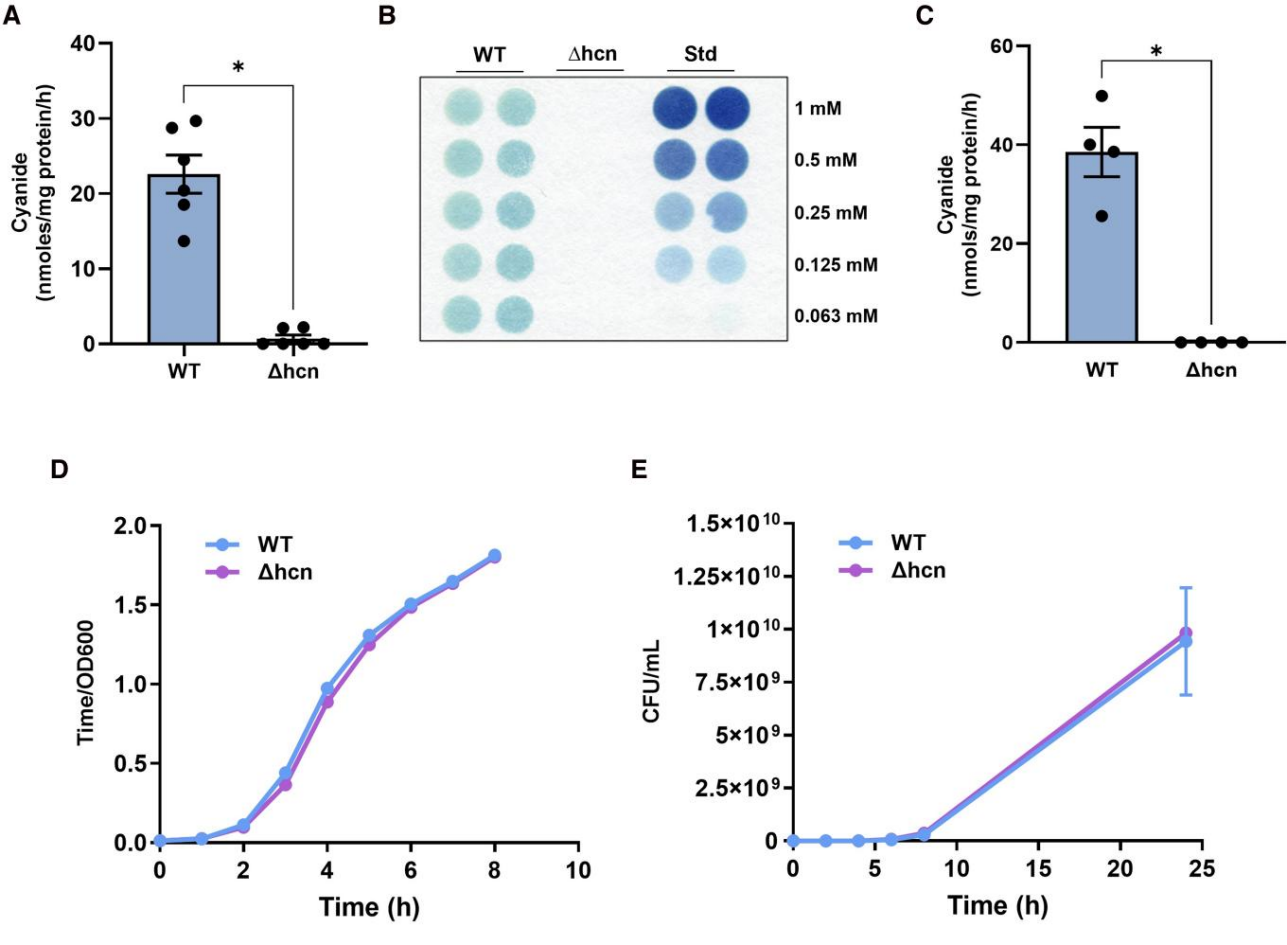
Cyanide production by WT or Δ*hcn P. aeruginosa*. *A*, Cyanide levels in culture supernatant of WT or Δ*hcn P. aeruginosa*, as measured using a CN-selective electrode. *B*, Cyanide release into the headspace by WT or Δ*hcn P. aeruginosa*, as measured by the Feigl–Anger paper method. *C*, HCN release quantification from WT or Δ*hcn P. aeruginosa*, using a colorimetric headspace assay based on Feigl–Anger paper. Growth curve assays for WT or Δ*hcn P. aeruginosa*, performed in (*D*) LB agar broth and (*E*) RPMI medium in the presence of 10% FBS. Experiments were performed on at least 4 biological replicates per group. Data are expressed as the mean ± SEM. *P < .05 indicates significant differences.

Wild-type *P. aeruginosa* produced measurable HCN, while the HCN synthase-null mutant (“Δhcn”) produced no detectable cyanide (Figure 1*A*–C). Polymerase chain reaction analysis confirmed the transposon insertion in the *hcnB* gene. The Δ*hcn* mutation did not affect bacterial growth (Figure 1*D* and 1*E*).

For bacteria-macrophage co-culture studies, we used U937 cells, a human monocytic cell line, differentiated with phorbol ester into macrophage-like cells. The differentiated cells showed decreased proliferation and increased adhesion, and formed microscopically observable small clusters (Supplementary Fig. 1). Differentiated cells showed a marked increase in their phagocytic activity and significant upregulation of monocytic (CD11b and CD14) and activation (CD80 and CD86) markers and cytokine production (Supplementary Fig. 1). The high phagocytic capacity of the differentiated cells was further confirmed using *E. coli* conjugated with pHrodo and fluorescent latex beads labeled with GFP (Supplementary Fig. 1).

**Figure 2.**
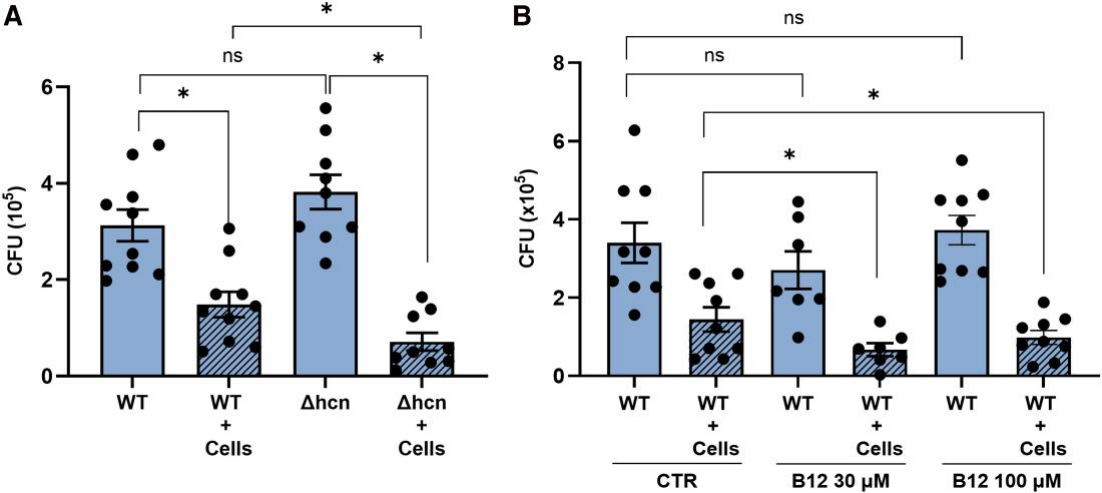
Killing activity of U937-derived macrophages against WT or Δ*hcn P. aeruginosa*. *A*, The cells were incubated with WT or Δ*hcn P. aeruginosa* (MOI 1). The experiments were performed in triplicates, and the average of colonies was used to calculate colony-forming units. *B*, Killing activity against WT *Pseudomonas* in the presence of vitamin B_12_ (30 or 100 µM). Experiments were performed on at least 8 biological replicates per group. Data are expressed as the mean ± SEM. *P < .05 indicates significant differences.

To determine whether HCN production influences bacterial killing, bacterial-macrophage co-cultures were performed and CFUs were measured. Significantly fewer bacteria survived when the Δ*hcn* mutant was co-cultured with macrophages compared with WT (Figure 2*A*). In the presence of U937 cells, WT bacterial counts were reduced to 45 ± 8% of control levels, whereas Δ*hcn* bacterial counts were reduced to 16 ± 5%, corresponding to approximately 64% reduction relative to WT (*P* < .05; Figure 2*A*). Similar effects were also observed in co-cultures of *Pseudomonas* with J774 murine macrophages: at 90 minutes of co-incubation, bacterial killing was higher (75 ± 6%) in the Δ*hcn* mutant co-cultures than in the WT controls (62 ± 7%; n = 8, *P* < .05). Moreover, at 3 hours of co-culturing in the presence of cells, WT bacterial counts were reduced to 11 ± 4% of control levels, whereas Δ*hcn* bacterial counts were reduced to 5 ± 1%, corresponding to a 56% reduction relative to WT (*P* < .05, n = 8).

Addition of the cyanide scavenger hydroxocobalamin (vitamin B_12_) to WT *Pseudomonas*—U937 co-cultures partially phenocopied the Δ*hcn* mutant: CFUs were reduced relative to untreated co-cultures (Figure 2*B*). Another HCN scavenger, THC had similar effects; at 30 µM, CFUs in the control (vehicle) group or the THC group were reduced by co-culture with the U937 macrophages by 50 ± 7% versus 79 ± 6%, respectively (n = 7, *P* < .01).

**Figure 3.**
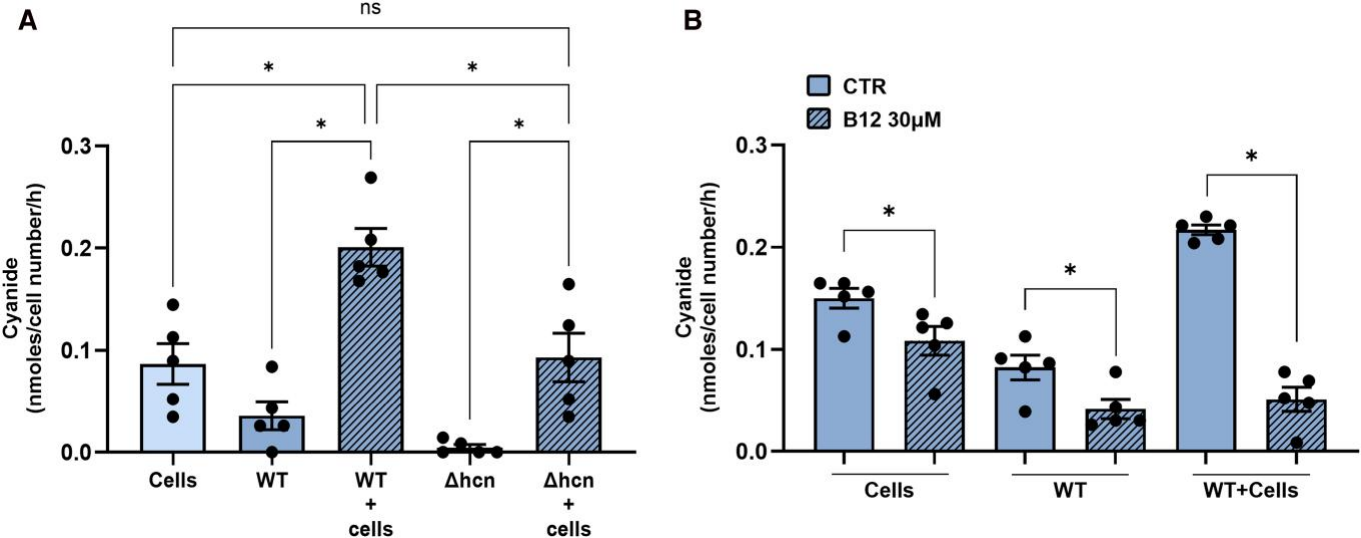
Cyanide detection in supernatant of U937-derived macrophages co-cultured with WT or Δ*hcn P. aeruginosa*. Cyanide detection was performed after 3 h incubation with the bacteria. *A*, Cyanide levels in cells without bacteria and cells in the presence of WT or Δ*hcn P. aeruginosa*. U937 cells (passages 4–6) were used. *B*, Cyanide levels in cells and in co-cultures of the cells with WT *P. aeruginosa* without (CTR) or with vitamin B_12_ (30 and 100 µM). U937 cells (passages 8–10) were used. Experiments were performed on at least 5 biological replicates per group. Data are expressed as the mean ± SEM. *P < .05 indicates significant differences.

U937 macrophages––in the absence of bacterial addition––generated detectable amounts of cyanide (Figure 3*A*), although––when normalized to cell counts or to cellular protein levels––*P. aeruginosa* was a drastically more potent producer of cyanide than the mammalian cells. For instance, mammalian cells secreted 4.4 ± 1.1 × 10^5^ nmoles cyanide per mg cell protein per hour, while bacteria secreted 1.8 ± 0.7 × 10^8^ nmoles, which corresponds to an approximately 400-fold higher production rate (n = 8, *P* < .001). Cells co-cultured with WT *P. aeruginosa* also produced cyanide, which was detectable in the supernatant (Figure 3*A*). As expected, Δ*hcn* bacteria generated no detectable cyanide (Figure 3*A*). Vitamin B_12_ (30 or 100 µM) reduced cyanide levels in the supernatant (Figure 3*B*).

**Figure 4.**
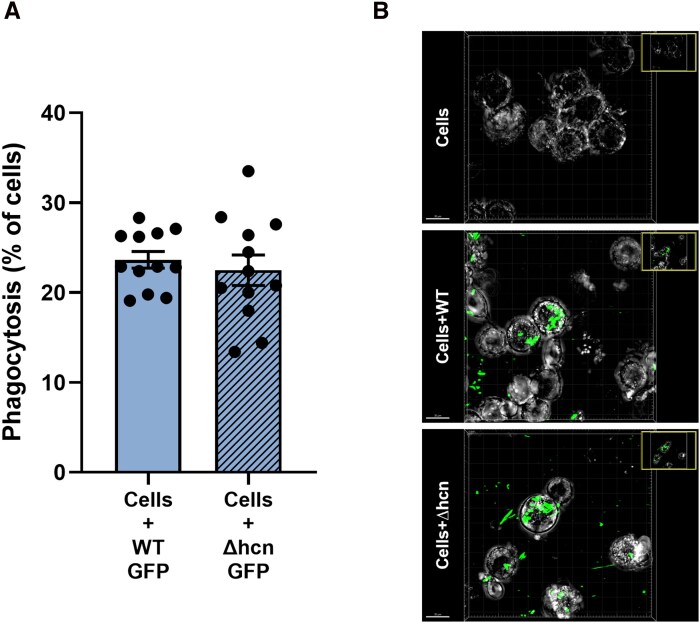
Phagocytosis of WT or Δ*hcn P. aeruginosa* by U937-derived macrophages. *A*, Phagocytic activity of cells after 3 h of incubation with GFP-labeled WT or Δ*hcn P. aeruginosa.* Experiments were performed on 12 biological replicates per group. Data are expressed as the mean ± SEM. No significant difference was observed between the 2 groups. *B*, Representative confocal images of GFP-labeled bacteria in the phagocytosis assay, to illustrate the presence of bacteria inside the mammalian cells. Images are representative and not used for quantification.

To determine whether cyanide affects bacterial uptake by the differentiated cells, we compared phagocytosis of WT versus Δ*hcn* bacteria (using GFP-labeled bacteria for visualization). U937 macrophages internalized both strains equally well (Figure 4*A* and 4*B*), indicating that the increased elimination of the Δ*hcn* bacteria was not due to increased phagocytosis.

**Figure 5.**
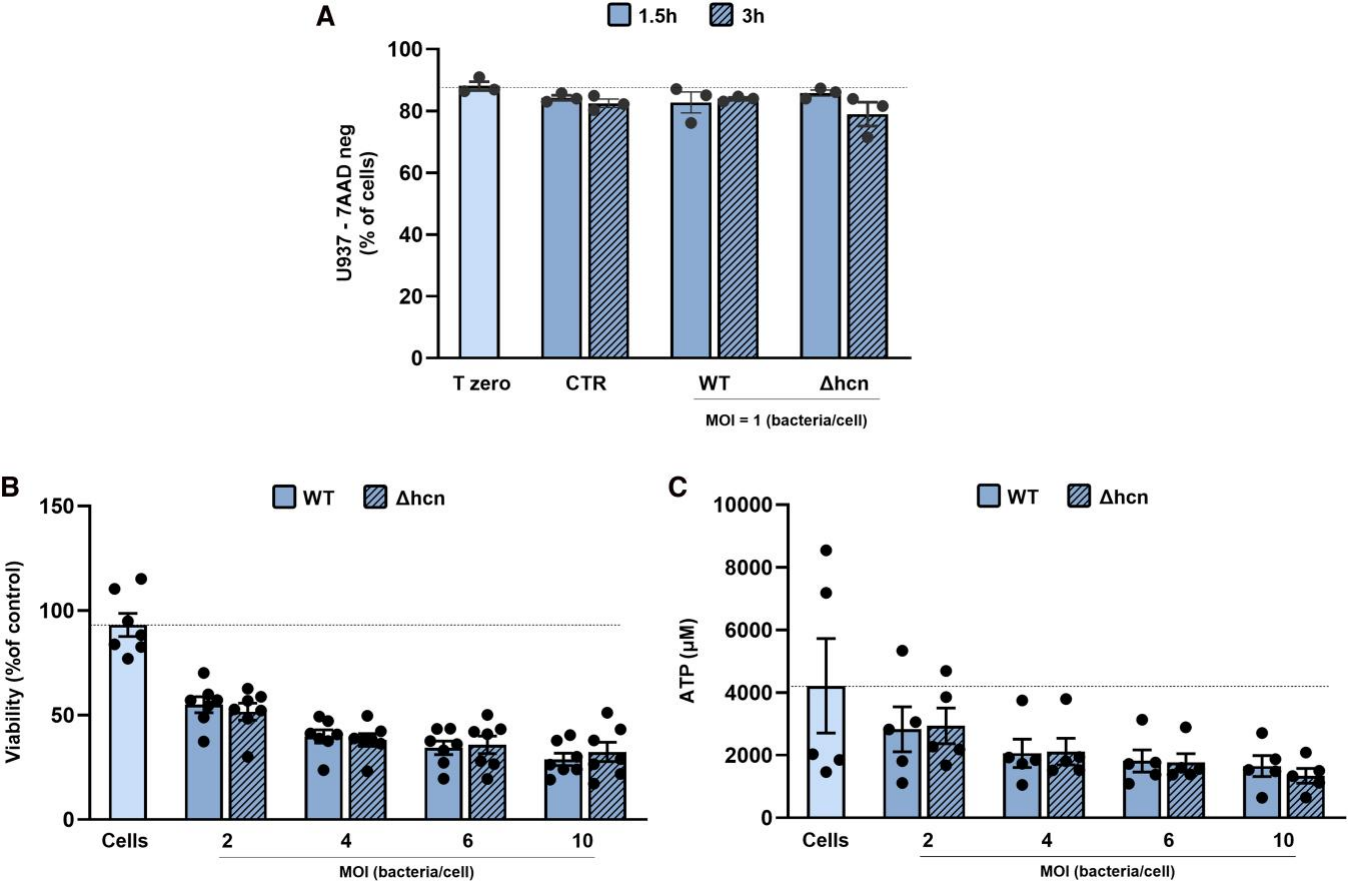
Viability, MTT reduction capacity, and ATP production in U937-derived macrophages infected with WT or Δ*hcn P. aeruginosa*. Cells were incubated with various amounts of WT or Δ*hcn P. aeruginosa* added to 96-well plates, for up to 3 h. *A*, Cell viability (identified by flow cytometry as 7-AAD negative cells) at 1.5 and 3 h after addition of *P. aeruginosa* (MOI 1); (*B*) MTT activity and (*C*) ATP levels, measured by luminescence (2–10 MOI bacteria/cells). Experiments were performed on at least 5 biological replicates per group. Data are expressed as the mean ± SEM. No significant difference was observed between the 2 groups.

We also compared the viability and ATP content of U937 macrophages after exposure to different amounts of WT or Δ*hcn* bacteria. The bacteria did not exert cytotoxic effects on the mammalian cells (Figure 5*A*), but reduced their mitochondrial respiratory activity (Figure 5*B*) and ATP content (Figure 5*C*). However, no significant differences were observed in these effects between WT versus Δ*hcn* bacteria (Figure 5*A–C*).

**Figure 6.**
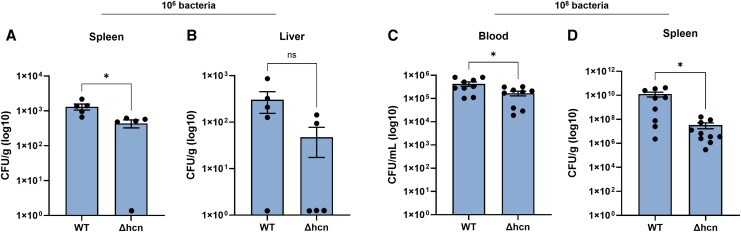
Bacterial clearance WT or Δ*hcn P. aeruginosa* in vivo. CFU counts in (*A*) spleen and (*B*) liver homogenates from mice inoculated with WT or Δ*hcn P. aeruginosa* (400 µL of 1 × 10^6^ bacteria) or in (*C*) blood and (*D*) spleen homogenates from mice inoculated with WT or Δ*hcn P. aeruginosa* (400 µL of 1 × 10^8^ bacteria). In the experiments using 1 × 10^6^ bacteria, no detectable CFUs were found in the blood (not shown). After 8 h of monitoring, animals were sacrificed, and blood, spleen, and liver tissue were collected. Homogenates were seeded in LB agar plates at various dilutions. The plates were incubated at 37°C, and the average number of colonies was used to calculate CFU/mL. Experiments were performed on 5 animals per group in the experiments utilizing 10^6^ bacteria and 9 animals per group in the experiments utilizing 10^8^ bacteria. Data are expressed as the mean ± SEM. *P < .05 indicates significant differences.

To evaluate the role of *P. aeruginosa–*derived cyanide to modulate bacterial elimination by the immune system in vivo, mice were injected with either WT or Δ*hcn P. aeruginosa* at 2 different bacterial loads (10^6^ and 10^8^ CFU). Eight hours postinfection, the mice infected with Δ*hcn Pseudomonas* had significantly lower bacterial loads in blood and spleen compared with mice infected with WT bacteria (Figure 6*A*–*D*).

## DISCUSSION

**Figure 7.**
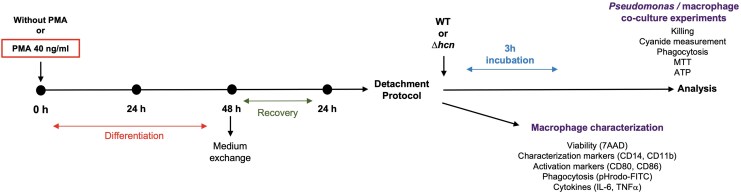
Schematic diagram of the experimental design.

The central finding of the current report is that HCN produced by *P. aeruginosa* can subvert the host's antimicrobial immune response. As shown in an in vitro set of experiments (Figure 7), wild-type *P. aeruginosa* was significantly more resistant to killing by macrophages in vitro. In addition, it was also associated with higher bacterial burden in vivo, compared with an HCN-deficient mutant strain. Thus, HCN generation contributes to *P. aeruginosa* pathogenesis by enabling the bacterium to evade innate immunity. This conclusion is supported by convergent genetic (Δhcn) and pharmacological (cyanide scavenging) approaches, both yielding consistent phenotypes. The above conclusion is also in line with prior studies showing that certain *P. aeruginosa* isolates produce sufficient HCN to functionally impair host organisms in various experimental settings using model systems (eg, *C. elegans* and *D. melanogaster*) [11–13].

Hydrogen cyanide thus joins a list of known *P. aeruginosa* virulence factors that can antagonize host defenses. Unlike proteases or exotoxins, HCN represents a volatile, diffusible metabolite with a unique mode of action. Our results may be relevant in the context of clinical observations linking *P. aeruginosa* HCN production to worse outcomes. In chronic respiratory infections such as those in CF patients, the importance of HCN generation by *P. aeruginosa* was proposed already 10 years ago [16–19]. *Pseudomonas*-derived HCN is likely a pathophysiologic contributor to lung damage, correlating negatively with pulmonary function [19]. Sputum from CF patients chronically colonized with *P. aeruginosa* has cyanide concentrations averaging approximately 70 µM and reaching up to 130 µM. In fact, *P. aeruginosa* infection in CF often creates microaerobic biofilm pockets, allowing local HCN accumulation to such levels. Importantly, patients with detectable sputum cyanide had significantly poorer lung function than those without detectable cyanide [20–27]. At these concentrations, HCN is cytostatic or cytotoxic to human cells [4, 5].

Thus, HCN production may confer an advantage to *P. aeruginosa* in the host and exacerbate disease severity—possibly by damaging host tissues and blunting immune clearance. In addition to CF, it is conceivable that the same mechanism may occur in other diseases where *P. aeruginosa* plays a pathogenetic role, including septicemia (sepsis and septic shock) and urinary tract infections. We hypothesize that HCN production may directly contribute to *P. aeruginosa* persistence and pathogenicity across these conditions, at least in part by hampering immune clearance.

It is well known that *P. aeruginosa* employs multiple immune evasion strategies. Our first working hypothesis was that bacterial HCN generation counteracts the phagocytic ability of the macrophages. However, direct measurements showed this was not the case: both WT and HCN-deficient *P. aeruginosa* were taken up by macrophages to a comparable degree. Our next hypothesis––based on HCN's well-known ability to inhibit mitochondrial complex IV, and thus inhibit oxidative phosphorylation, ATP generation, and suppress cellular viability and various cellular functions [5]––was that HCN generation by *P. aeruginosa* may impair macrophage bioenergetics. However, once again, the data showed this was not the case: both WT and HCN-deficient *P. aeruginosa* suppressed macrophage MTT activity (a parameter of mitochondrial function) and intracellular ATP levels to a comparable degree.

What, then, is the mechanistic basis of HCN's ability to suppress the killing capacity of the macrophages? HCN's toxicity is well characterized: it binds metalloenzymes (particularly those with heme iron) [5]. One possible hypothesis is that cyanide inhibits the activity of essential metalloenzyme(s), perhaps those that produce reactive oxygen or chlorinating species (eg, superoxide, hydrogen peroxide, and hypochlorous acid) involved in bacterial killing. Indeed, recent work demonstrated that *P. aeruginosa*–derived HCN acts as a scavenger of HOCl, neutralizing its toxic effects and protecting the bacteria from oxidative damage [26]. In addition to directly consuming HOCl, cyanide might inhibit reactive oxygen species (ROS) generation upstream: the reduced nicotinamide adenine dinucleotide phosphate (NADPH) oxidase complex itself contains heme prosthetic groups (in cytochrome b558) and other redox centers could be inhibited by cyanide [5, 27]. Indeed, significant prior literature indicates that cyanide suppresses the oxidative burst response of immune cells [28–35]. Dysregulation of NADPH oxidase activity due to cyanide would impair the formation of reactive oxygen species needed to kill engulfed bacteria. Cyanide might also interfere with myeloperoxidase (MPO), since MPO is a heme enzyme—binding of cyanide to MPO's heme could inhibit the enzyme's production of HOCl from hydrogen peroxide [5, 33–35]. While the precise molecular targets of cyanide in our *P. aeruginosa–*macrophage co-culture system remain to be elucidated, the net effect observed in our study is clear: HCN renders macrophages significantly less effective at killing *P. aeruginosa*. The magnitude of the effect––based on the in vivo findings––is substantial; CFU counts in the spleen and lung were more than 60%–95% lower with HCN-deficient bacteria than with WT controls; in the in vitro experiments, macrophage-mediated killing was approximately 2-times more efficient with HCN-deficient bacteria. The response with vitamin B_12_ was less pronounced than the difference between WT and HCN-deficient bacteria, likely because the vitamin only partially reduced cyanide concentrations in our co-culture system.

Our findings on cyanide evoke interesting parallels with another small, gaseous molecule produced by bacteria: hydrogen sulfide (H_2_S). In recent years, H_2_S has emerged as a bacterial defense factor against both antibiotics and immune attack. Many pathogenic bacteria (including *E. coli* and *Staphylococcus aureus*) endogenously generate H_2_S, which can protect them from oxidative stress [36, 37]. We showed that bacterial H_2_S increases resistance to macrophage and neutrophil killing and that inhibiting H_2_S production or deleting H_2_S-generating genes renders bacteria significantly more susceptible to immune clearance [23]. In mouse models, H_2_S-deficient bacteria were cleared more rapidly by the immune system [23], mirroring what we observed with HCN-deficient *P. aeruginosa*. Thus, cyanide generation in *P. aeruginosa* may play a role analogous to H_2_S in other pathogens—both function as endogenously produced gasotransmitters that enhance bacterial survival under host-induced oxidative stress. Mechanistically, there are similarities: like cyanide, H_2_S can inhibit cytochrome c oxidase and other heme enzymes at high concentrations, leading to transient metabolic shutdown in host cells. In this context, it will be interesting to determine whether the observed inhibition of host mitochondrial function by *Pseudomonas* may be related to bacterial H_2_S or NO generation––as our results show that it is not related to HCN, since the degree of inhibition of mitochondrial MTT conversion and ATP generation was comparable in WT and cyanide-deficient *Pseudomonas*/host co-cultures.

H_2_S is also a potent reductant that can quench reactive oxygen species (eg, it can directly scavenge peroxides and peroxynitrite) thereby it may protect bacteria from the oxidative burst [36, 37]. Notably, inhibiting bacterial H_2_S synthesis increases phagocytic killing by leukocytes [23], just as our HCN-knockout strain was more readily eliminated by macrophages. These convergent findings underscore a broader principle: bacteria produce small inorganic molecules (H_2_S, HCN, nitric oxide, etc.) as defensive virulence factors to subvert host immune mechanisms. It will be interesting to explore, for instance, if the bacterial H_2_S and HCN systems interact in the host, especially since the mitochondrial enzyme rhodanese (thiosulfate sulfurtransferase [TST]) plays roles in the clearance of both HCN and H_2_S [5, 37]. The delineation of such potential interactions remains to be performed in follow-up experiments.

It should be pointed out that despite many similarities, HCN and H_2_S also have important differences. Almost all bacteria have pathways that generate H_2_S (often as a by-product of cysteine metabolism), making H_2_S a widespread and sometimes constitutive molecule, whereas HCN production is relatively rare—largely confined to *P. aeruginosa* and a few other species. *Pseudomonas aeruginosa* invests in a dedicated HCN biosynthetic operon and even an HCN-resistant respiratory chain, indicating cyanide's significance in its ecology and infection strategy. From the pathogen's perspective, both HCN and H_2_S confer resilience against oxidative killing. Since *P. aeruginosa* is a known H_2_S producer [38–40], it will be interesting to explore in the future if H_2_S and HCN play cooperative, perhaps additive or synergistic roles in evading host immune responses.

From a translational perspective, our work raises the prospect of targeting bacterial HCN production or function as a novel therapeutic or adjunctive strategy in *P. aeruginosa* infections. Novel approaches to target this bacterial strain are clinically relevant due to the pathogenicity of this bacterial strain and its known tendency to develop antibiotic resistance. Given that HCN enables immune evasion, strategies that neutralize cyanide could restore the effectiveness of host immunity and improve infection outcomes. One potential approach is the use of already-known cyanide scavengers. For example, hydroxocobalamin (vitamin B_12a_) binds cyanide with high affinity to form cyanocobalamin, and it is used clinically to treat cyanide poisoning due to smoke inhalation. It has a favorable safety profile and could, in theory, be repurposed. Cobinamide derivatives, such as THC used in the current study, have a higher cyanide scavenging affinity and have been used in various experimental settings in vitro and in vivo previously [41–44] and may be potentially clinically translatable. Another potential approach may be to inhibit HCN synthesis at the bacterial end—that is, target the HCN synthase enzyme or its regulation. Hydrogen cyanide synthase requires glycine as a substrate; molecules that mimic glycine's structure or otherwise block the HCN–ABC enzymatic complex could reduce cyanide output. Cyanide neutralization would not be considered a classical “antibiotic” approach but rather a modulatory approach that *enhances the ability of the host system to eliminate the pathogen*. Conceptually, this is similar to how novel inhibitors of bacterial CSE or CBS (H_2_S-producing enzymes) [23, 38] enhance the elimination of bacteria by the host immune system.

## Supplementary Material

jiag244_Supplementary_Data

## Supplementary Data

Supplementary materials are available at *The Journal of Infectious Diseases* online (http://jid.oxfordjournals.org/). Supplementary materials consist of data provided by the author that are published to benefit the reader. The posted materials are not copyedited. The contents of all supplementary data are the sole responsibility of the authors. Questions or messages regarding errors should be addressed to the author.
