# Supplementary material for: Hydrogen Cyanide Generation by Pseudomonas aeruginosa Blunts the Host Innate Immune Response
Source: J Infect Dis. 2026 May 6;234(1):e141–50. doi: 10.1093/infdis/jiag244 (PMC13431797; doi:10.1093/infdis/jiag244)
Supplement: jiag244_Supplementary_Data [file jiag244_supplementary_data.pdf]

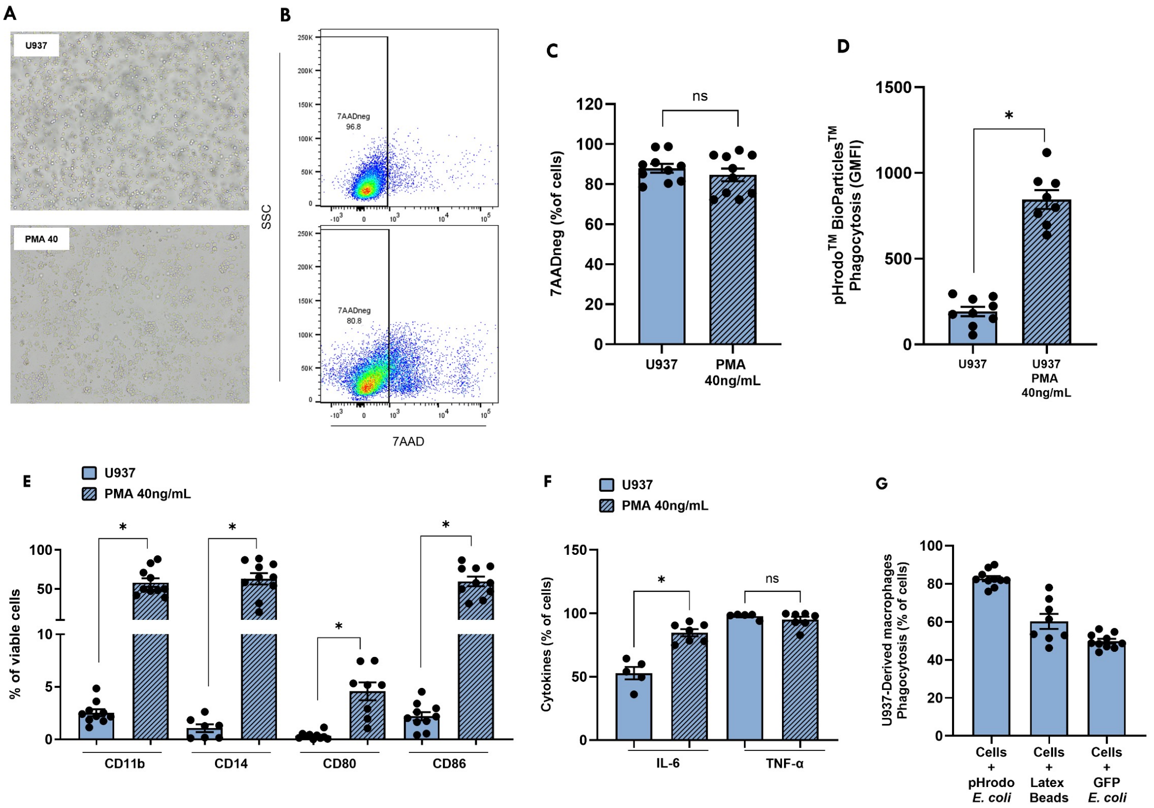


**Supplemental Figure 1. Macrophage differentiation after 48 h incubation with PMA.** (**A**) Representative microscope images (objective 40X) of cells before and after incubation with PMA (40 ng/ml). (**B**) Representative dot plots of analysis of 7-AAD negative population (viable cells). (**C**) Percent of viability of cells by flow cytometry. (**D**) Phagocytotic capacity of U937 cells, under baseline conditions and after differentiation with PMA. (**E**) Detection of various activation markers of U937 cells, under baseline conditions and after differentiation with PMA. (**F**) Intracellular cytokine production of U937 cells, under baseline conditions and after differentiation with PMA. (**G**) Phagocytosis activity of U937-derived macrophages incubated with *E. coli* pHrodo-labeled, Latex beads yellow green-labeled or GFP-labeled *E. coli*. Experiments were performed on at least 5 biological replicates per group. Data are expressed as the mean ± SEM. *P < 0.05 indicates significant differences.
